# Double–blind control of the data manager doesn't have any impact on data entry reliability and should be considered as an avoidable cost

**DOI:** 10.1186/1471-2288-8-66

**Published:** 2008-10-20

**Authors:** Davide Mauri, Vasiliki Karampoiki, Jacopo Mauri, Konstantinos Kamposioras, Georgios Alexiou, Georgios Ferentinos, Lamprini Tsali, Ioanna Karathanasi, Christina Peponi, the SESy_Europe task Force

**Affiliations:** 1 PACMeR sections of Oncology and Public Health, Athens, Greece; 2 Dept. Med. Oncology, Papageorgiou Hospital, Thessaloniki, Greece; 3 PACMeR, Engineering and Development section, Milan, Italy

## Abstract

**Background:**

Database systems have been developed to store data from large medical trials and survey studies. However, a reliable data storage system does not guarantee data entering reliability.

We aimed to evaluate if double-blind control of the data manager might have any effect on data-reliability. Our secondary aim was to assess the influence of the inserting position in the insertion-sheet on data-entry accuracy and the effectiveness of electronic controls in identifying data-entering mistakes.

**Methods:**

A cross-sectional survey and single data-manager data entry.

Data from PACMeR_02 survey, which had been conducted within a framework of the SESy-Europe project (PACMeR_01.4), were used as substrate for this study. We analyzed the electronic storage of 6446 medical charts. We structured data insertion in four sequential phases. After each phase, the data stored in the database were tested in order to detect unreliable entries through both computerized and manual random control. Control was provided in a double blind fashion.

**Results:**

Double-blind control of the data manager didn't improve data entry reliability. Entries near the end of the insertion sheet were correlated with a larger number of mistakes. Data entry monitoring by electronic-control was statistically more effective than hand-searching of randomly selected medical records.

**Conclusion:**

Double-blind control of the data manager should be considered an avoidable cost. Electronic-control for monitoring of data-entry reliability is suggested.

## Background

Large survey studies are important for public health policy making and to improve the effectiveness of interventions. Database systems and electronic networks have been developed to render surveys more manageable by providing data storing and analysis [1,2]. Data standardization and accuracy, as well as secure storage are of particular importance in multi-center studies. However, the availability of reliable electronic systems is not enough to guarantee the validity of population-based cross-sectional studies. Indeed, the relevance of a medical survey is largely dependent on two main steps: the quality of data collection in the medical-charts and the fidelity of data transferring from the charts to the electronic system. Any weakness in these two stages will invalidate the study [3-7].

The present study is focused on data-entering reliability. Many techniques, such as combo-boxes, filters that prevent fields being in logical contradiction to other values and the involvement of specialized data-managers or of a single data-manager have been successfully introduced to reduce transcriptional mistakes. However, the process of data entering could still represents a problem for data reliability.

In the present study (SESy-Europe project), conducted within a framework of a nationwide Hellenic survey of cancer screening assessment, we set out to evaluate if a double blind control of the inserted data might have a clear effect on the data-management, thus reducing mistakes during data entering. Furthermore, we evaluated if the inserting position in the insertion-sheet has any impact on occurrence of mistakes. Furthermore, we investigated whether an electronic identification of high-risk insertions might be more sensitive than random control of the questionnaires in identifying data-entering mistakes.

## Methods

This study is a part of the Screening Evaluation System Europe (SESy-Europe) project, also known as the PACMeR_01.04 project because it is organized by the Panhellenic Association for Continual Medical Research. SESy-Europe project is a multinational study involving fourteen centres in ten European Nations and tailored to the development of a multilanguage database able to bridge European countries in cancer screening monitoring policy.

In this study, SESy-Europe project has used data coming from medical charts (questionnaires) of a Greek survey that aimed at the evaluation of Hellenic cancer preventive and screening practices (PACMeR_02 study). Details on PACMeR_02 study have been already reported [8,9].

The project was ethically approved by PACMeR's Scientific Committee (protocol number 08_020720) and conformed to the ethical guidelines of the 1975's Declaration of Helsinki.

Data coming from 6446 medical charts (3462 female, 2984 male) and their electronic storing constituted the substrate of the analyses.

### Data entering and database

Data storing had been assured by SESy_Europe Database [10,11]. Despite the fact that the database has been tested for data-safety of insertion from multi-centric data-management [12], in this study section all data were inserted by a single data-manager. This has been reported to reduce the inter-data manager errors and facilitate analyses by avoiding data-manager related bias [13].

### Study design and blinding

Data insertion had been conducted in four chronologically sequential phases. Each phase constituted of three stages: 1) data entering, 2) control applied to inserted data, 3) correction of mistakes.

During the phase I were recorded and controlled data from the first 325,773 questionnaires. Successively in the phase II were recorded and controlled data from 151,734 questionnaire. Sequentially data from 145,401 and 107,286 questionnaires were recorded and controlled during the phase III and the phase IV respectively.

Data manager could not progress to the next phase of data entering, until all the previous phase procedures (stage 1,2,3) had been concluded. Details for each stage are provided below:

### First stage (data entering)

all data coming from a definite number of medical-charts was recorded in an established peripheral unit of the database (Nafpaktos, Greece).

### Second stage (controls applied to inserted data)

The recorded data were electronically sent to the Central unit of the database (Ioannina, Greece) and then transferred to an external commission for electronic control (Milan, Italy). At the same time the registered medical charts were sent to the questionnaires' collection center (Ioannina, Greece) and then to the PACMeR archive (Lixouri Hospital, Greece). Neither the data manager operating in the peripheral unit (Nafpaktos), nor the control units (Milan and Lixouri) were aware of each other, thus assuring that the study was blind.

Data that entered the data-base underwent the following two analyses:

A. Computerized controls for possible unreliable data (by electronic filters e.g.: height < 140 or > 195 cm, weight < 40 or > 120 kg, age at first parturition < 18 or > 40, BMI < 17 or > 41 etc.), [Milan]. [see additional file 1]

B. Random controls of 200 medical records (randomization by table of random numbers), [Ioannina]

We defined as *potential mistakes *all medical records flagged either by computerized controls (A) or by random selection (B). Potential mistakes triggered hand-searching in hard copies to validate the correspondence between the data contained in the medical records and those in the database. Non-corresponding data were considered *real mistakes*. Conversely corresponding data were identified as *false positive*. Lists of potential and real mistakes were thereafter registered for statistical analyses.

### Third stage (corrections of mistakes)

A dedicated operator went to the peripheral unit to present the list of real mistakes to the data manager and discuss the related insertions. The same operator was crucial to assure that the data manager in the peripheral unit could not progress to new insertions, until all the *real mistakes *registered during controls for the previous phase had been corrected and discussed. The operator was instructed to change the data-base ID code of the peripheral unit prior to any new phase of the study for that purpose. The ID code identifies the peripheral unit and the phase of insertion for each electronic record.

### Insertion-sheet

considering that the position of entry in the insertion-sheet might influence the rate of mistakes (e.g., data entering errors from the last insertion field of a long insertion-sheet), we recorded the proportion of real insertion mistakes at the beginning and at the end of the insertion sheet. Therefore, the parameters *age *and *weight *at 4^th^, 5^th ^insertion position, respectively, were compared to the parameters *age at marriage *and *age at first sexual intercourse *at insertion positions114 and 115 respectively.

### Outcomes

we set out:

1. To estimate if the double-blind control of the inserted data and the following corrections might have any effect on the data-manager, reducing mistakes during successive phases of data-entering.

2. To investigate if the position in the insertion sheet has any impact on mistakes occurrence during data-entering.

3. To examine differences in sensitivity for detection of data-entering mistakes by comparing the results obtained analyzing randomly selected insertion sheets against those identified by computerized filters for unreliable data.

Analyses were performed in Intercooled Stata 8.2 (Stata Corp, College Station TX, USA) using chi-square, Pearson chi-square and the *metareg *module. Unless otherwise specified, all statistical tests are two-tailed and statistical significance is set at p < 0.05.

## Results

### Population and insertions

PACMeR_02 surveyed 6446 individuals (2984 males,3462 females) for a total of 730,194 insertions were registered in the central table of the database (362,604 for females and 260,304 males respectively). The exact numbers of insertions per phase and for all analyzed fields are reported in Table 1.

**Table 1:** Number of insertions per each parameter analyzed for each phase of the study.

Parameter	field type	phase I	phase II	phase III	phase IV	Total insertions
All	-	325,773	151,734	145,401	107,286	730,194
Male	-	140,658	641,58	55,488	44,064	260,304
Females	-	185,115	87,576	89,913	63,222	362,604
Age	n	2,884	1,341	1,275	946	6,446
Age at first parturition^#^	n	1,505	712	731	514	3,462
Age marriage^#^	n	1,505	712	731	514	3,462
Education	c	2,884	1,341	1,275	946	6,446
Family position	c	2,884	1,341	1,275	946	6,446
First sexual intercourse^#^	n	1,505	712	731	514	3,462
Height	n	2,884	1,341	1,275	946	6,446
Insurance	c	2,884	1,341	1,275	946	6,446
Provenance	c	2,884	1,341	1,275	946	6,446
Sons	n	2,884	1,341	1,275	946	6,446
Number of parturitions ^#^	n	1,505	712	731	514	3,462
Urban community	c	2,884	1,341	1,275	946	6,446
Weight	n	2,884	1,341	1,275	946	6,446

Insertions concerning only females (#), insertion-fields type: combo-boxes (c) and numeric (n).

The number of "potential mistakes" identified by electronic controls (for each parameter analyzed per each phase) and the number of "real mistakes" encountered during the hand-searching check of "potentially mistakes" on medical charts are reported in Table 2.

**Table 2:** potential mistakes (pM) found in the electronic check and relative number of real mistakes (M) encountered in manual hard-copy check.

	Phase I		Phase II		Phase III		Phase IV	
	
	pM	M	pM	M	Pm	M	pM	M
Age	216	3	19	3	80	1	101	4
Age first parturition	267	4	30	1	37	2	22	1
Age marriage	153	1	65	3	75	0	43	1
Education	210	0	NA	NA	71	3	44	20
Family position	210	0	NA	NA	11	2	13	2
First sexual intercourse	205	1	82	2	101	0	78	1
Height	728	28	54	7	26	2	19	1
Insurance	210	4	NA	NA	35	22	36	3
Provenance	210	1	NA	NA	15	4	28	3
Sons	215	11	NA	NA	4	0	4	1
N of births (parturition)	11	7	3	3	3	1	4	0
Weight	690	30	41	4	32	6	26	1

### Outcome analyses

#### Effect of double blind control on data manager

Double-blind control and mistakes correction has not been found to have any benefit on data entering reliability. The proportion of mistakes in the four phases did not show a statistically significant difference (p = 0.66). On the contrary, meta-regression analysis by phase showed a trend for augmenting the risk of producing mistakes at each successive phase by 1.07, but also this was far from being statistically significant p = 0.27. These results were also confirmed when we calculated the risk ratio for data-entry mistakes in phase I (RR = 1.0) *vs. *each other phase (phase II RR = 1.082 p = 0.74; phase III RR = 1.059 p = 0.76; phase IV RR = 1.277 p = 0.21).

#### Position in the insertion-sheet

We found that parameter position in the insertion sheet plays a major role in mistake occurrence (real mistakes); with last insertions being statistically associated with higher rate of mistakes than the insertions at the beginning. This was evident during each phase of the study for any type of control considered (electronic or random selection). Proportion of mistakes observed in last insertion fields was notably lower for combo-boxes than those for numerical values. Table 3

**Table 3:** For electronic-check the analyses compared the proportion of real mistakes vs. the proportion of potential mistakes.

	Phase	Insertion fields position		p.
	
		Initial (%)	End (%)	
Random selection of questionnaires	I	0,55	3,64	0.0026
	II	3,4	11,66	0.0210
	III	0	6,25	0.0008
	IV	1,65	3,93	< 0.001
				
Electronic Check	I	11,8	15,7	< 0.001
	II	2,23	10,3	< 0.001
	III	4,39	12,03	< 0.001
	IV	6,71	11,77	< 0.001

For random selection of questionnaires we evaluated the number of mistakes encountered vs. the number of questionnaire randomized for each field controlled.

#### Random vs. electronic check

When electronic control was compared against the random selection of questionnaires, it was found to be statistically more effective in evidencing mistakes (real mistakes) in two of the three parameters analyzed: "Age" 1/800 vs. 11/416 *p *< 0.001, "number of children" 9/800 vs. 12/223 *p *< 0.001. Filter used for "age at marriage" produced a large number of false positive and displayed a positive trend but did not reach statistical significance (15/424 vs. 5/336 *p *= 0.080).

## Discussion and conclusion

Large research projects offer significant advantages but there is always a problem concerning data collection and processing. It is important to ensure that information is entered into the database consistently and accurately [15,16]. Our study evaluated some methods for controlling data-entering. While modern data-entry technologies have greatly reduced entry errors by use of quality control mechanisms [4], even a small proportion of mistakes can have a great impact on a study's results. Inadvertent random and systemic errors introduced into datasets and their manipulation are well-defined sources of bias in the statistical evaluation of clinical trials. Recently, Marks suggested the elimination of paper from clinical data capture and the use of computers from the start in order to maximize data-reliability [14]. However, elimination of hard-copies is usually not possible, thus many efforts had been done to reduce data-entering mistakes.

Besides studying electronic control in data-entering, the consequence of double data entry compared to single entry had been investigated in a double-blind setting, but data entry error rates were not significantly reduced [13]. This result may be explained by the fact that a single data-manager may reduce the inter data-manager bias and since errors are systematic they will be more easily identified than in a double data entry setting. The use of a single data-manager is important also from economical standpoint since the cost of a single data-manager was notably lower than a double-blind control system with double data entry [13].

For all the above reasons, our study had been performed by a single data-manager and presents the novelty to test not only for the impact of a double-blind control but also for the sequential (by phase) educational sessions on data-entry mistakes, as well. While it was hypothesized that this high quality controls might reduce the rate of insertion mistakes, our study showed that this combined approach did not seem to be effective and its use is therefore not recommended. Not only there was absence of improved data-entry reliability, but the double blind control sessions were associated with interruptions in the workflow of the data-manager (time and working-hours lost), useless employment of personnel and waste of resources and consequently increased expenditures. These results might be partially explained by the fact that well-trained and well-monitored data entry staffs are not the weakest link in the data management chain [17].

Our study also suggests that the position in the insertion field plays a very important role in the proportion of mistakes. The last positions are associated with more mistakes than the initial ones, especially when numeric fields are considered. This has been attributed to the fatigue of the data-manager when questionnaires have too many entries. These results therefore suggest that to create more effective questionnaires the most important information should be collected in the first fields, the number of insertion-fields per insertion-sheet should be reduced and combo-boxes or text-boxes should be used instead of fields with direct numerical insertion (especially in the last part of the questionnaire).

Furthermore, we found that electronic controls for insertion mistakes are more effective than manual searching of randomly selected medical charts: electronic search is far simpler; it is associated with lower time loss and reduced need of personnel. Its use is therefore recommended in quality-control for data-storing processes.

One limitation of this study is that it was based on a single data manager, thus it is difficult to generalize our conclusions. However, it should be remembered that the decision to use a single data-manager was introduced to improve data entry-reliability by reducing inter data-manager bias [13]. Keeping in mind these limitations, we nevertheless believe that our conclusions are useful and may help guide data-management decisions and improve data-entering reliability.

## Competing interests

The authors declare that they have no competing interests.

## Authors' contributions

DM conceived of the study, and participated in its design and coordination and drafted the manuscript. VK participated in the study design, data collection and drafted the manuscript. JM and GF participated in the study design and were the responsible for the electronic-controls and statistical analyses. KK and GA were responsible for the hand-searching (manual controls). LT participated in study design and coordination and drafted the manuscript. IK participated in the study design and questionnaires collection. CP participated as single data-manager. All authors read and approved the final manuscript.

## Note

SESy_Europe task Force: Francisco Javier Rivas Flores *(San Rafael Hospital, Madrid -Spain-)*; Hilal Altinoz *(SSK Sureyyapasa, Thoracic Disease Center, Istanbul -Turkey-)*; Marzanna Chojnacka *(Maria Sklodowska-Curie Memorial Cancer Center, Warsaw -Poland-) *Irini Karentzou *(University school of Medicine, Cologne -Germany-)*; Camelia Colichi *(Institute of Oncology, Bucharest -Romania-) *Tamara Oxiuzova and Eleni Kanavoura *(University school of medicine, Ioannina -Greece-)*; Berta Adelaide Maia da Silva Alves de Sousa *(Portuguese Oncology Institute IPOPFG-EPE, Porto -Portugal-)*; Diana Ivanova *(PACMeR, Athens -Greece-)*, Mario Dambrosio *(Multimedica Hospital, Milan -Italy-)*.

## Pre-publication history

The pre-publication history for this paper can be accessed here:



## Supplementary Material

Additional file 1 **electronic controls.** The data provided represent the methodology used and the filters employed in electronic controls. Click here for file
