# Supplementary material for: Double–blind control of the data manager doesn't have any impact on data entry reliability and should be considered as an avoidable cost
Source: BMC Med Res Methodol. 2008 Oct 20;8:66. doi: 10.1186/1471-2288-8-66 (PMC2596166; doi:10.1186/1471-2288-8-66)

# Checks on Database

## Structure and short description

The checks on database are grouped in a file “Checks.mdb” which contents the links to all the tables of the project: tables containing the stored data and the tables containing the menus, codes and so on (functional tables).

Queries are structured in order to help the operator to show particular records (potential mistakes) thanks to tailor-made filters. Each filter is aimed to select from the whole database the records which show values which are out of standard or out average. The number of fields of the whole database for which this kind of check can be applied is only limited to the only ones in which could be evident a possible data – entry mistake (age, BMI, ecc)..

For this reason the list of records whose values exceed the defined warning – limit are controlled punctually comparing the data inserted with the one written on the original questionaries filled by each doctor.

In the next pages for each query is reported

- A basic structure
- A short description
- A sight of the structure of the query


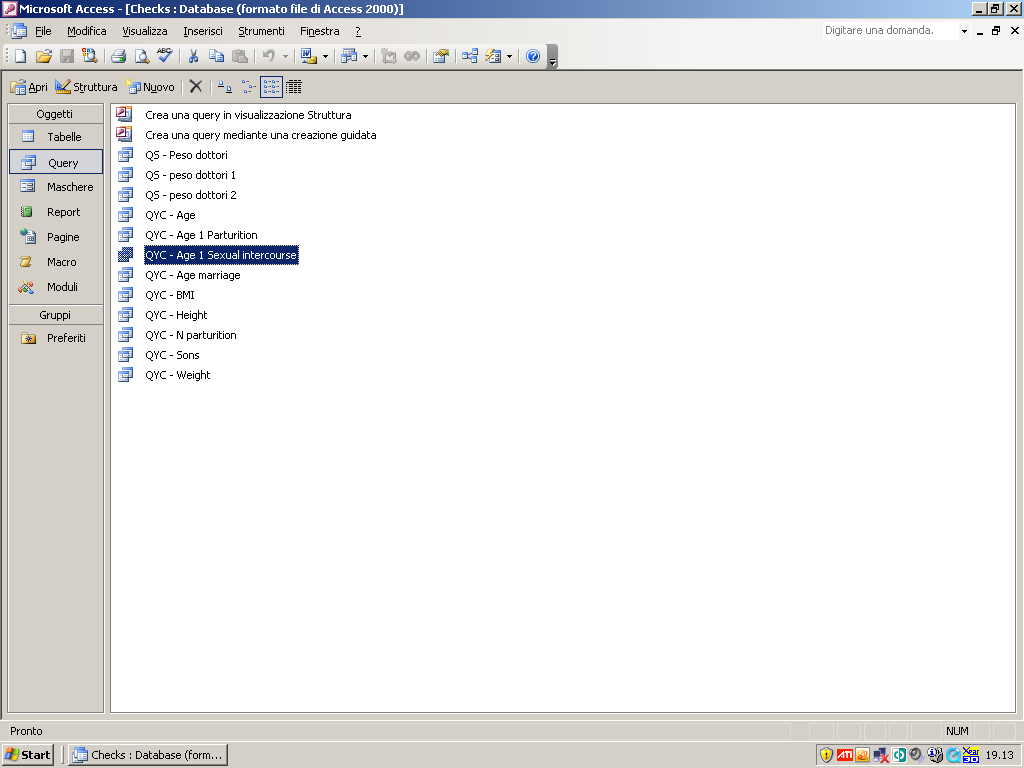

The description of each filter will be described in the next pages.

## Explication

### Age

QUERY NAME QYC - Age

FROM TABEL ST Cancer screening

SELECTED FIELD Age

FILTER: Age < 40 OR > 80

OTHER SWHOWN FIELDS - - -


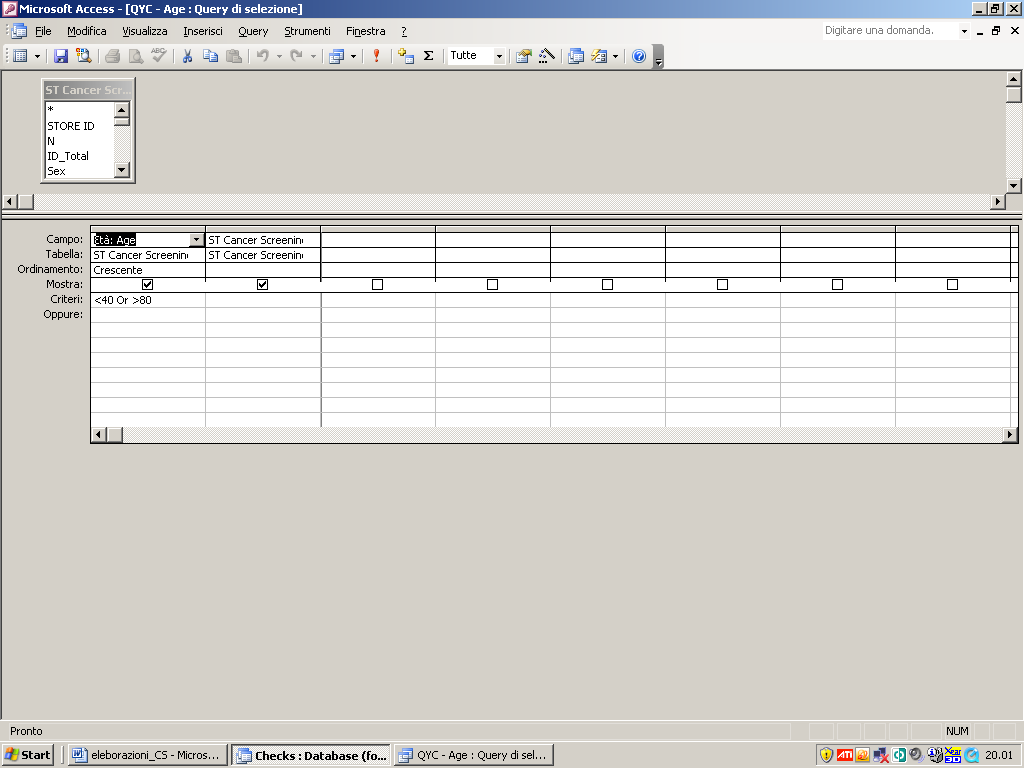


### Age First Parturition

QUERY NAME QYC – Age 1 Parturition

FROM TABEL ST Cancer screening

SELECTED FIELD Age first parturition

FILTER: Age < 18 OR > 40

OTHER SWHOWN FIELDS Sex


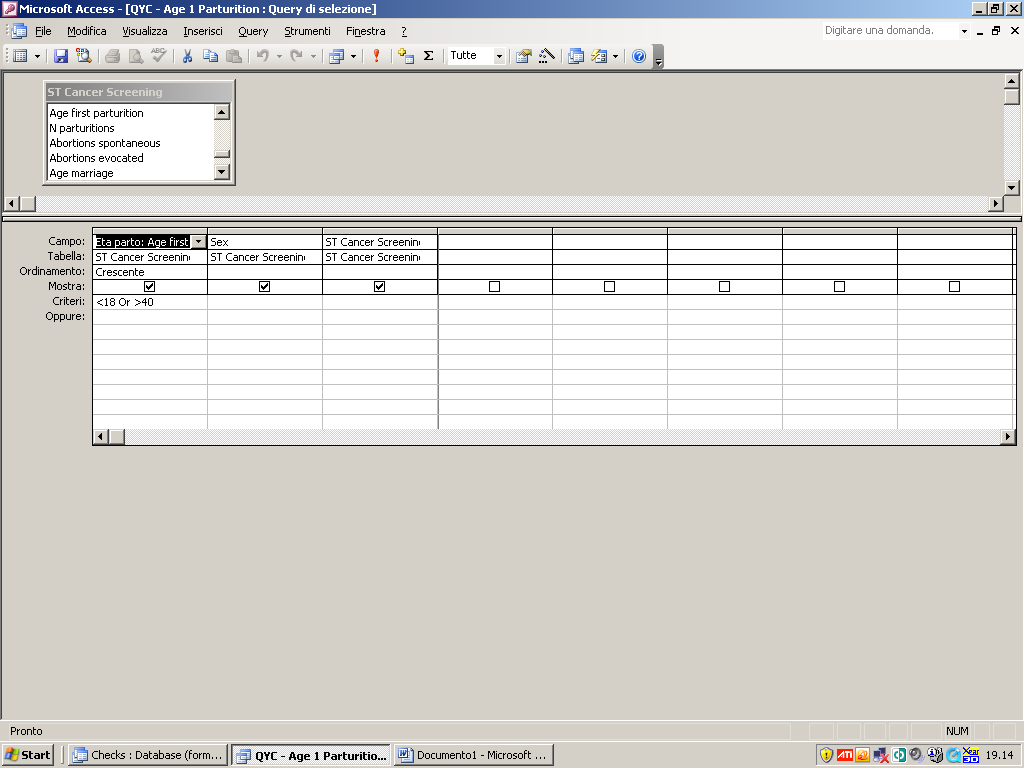


The first parturitions over 40 years and under 18 have can be a data – entry – mistake.

### The field “sex” is shown in order to verify if there hasn’t been a mistake in the data entry. (Male is infact an impossible value)

### Age First Sexual Intercourse

QUERY NAME QYC – Age 1 Sexual Intercourse

FROM TABEL ST Cancer screening

SELECTED FIELD Age sexual intercourse

FILTER: Age >0 AND < 18 OR > 40

OTHER SWHOWN FIELDS Sex


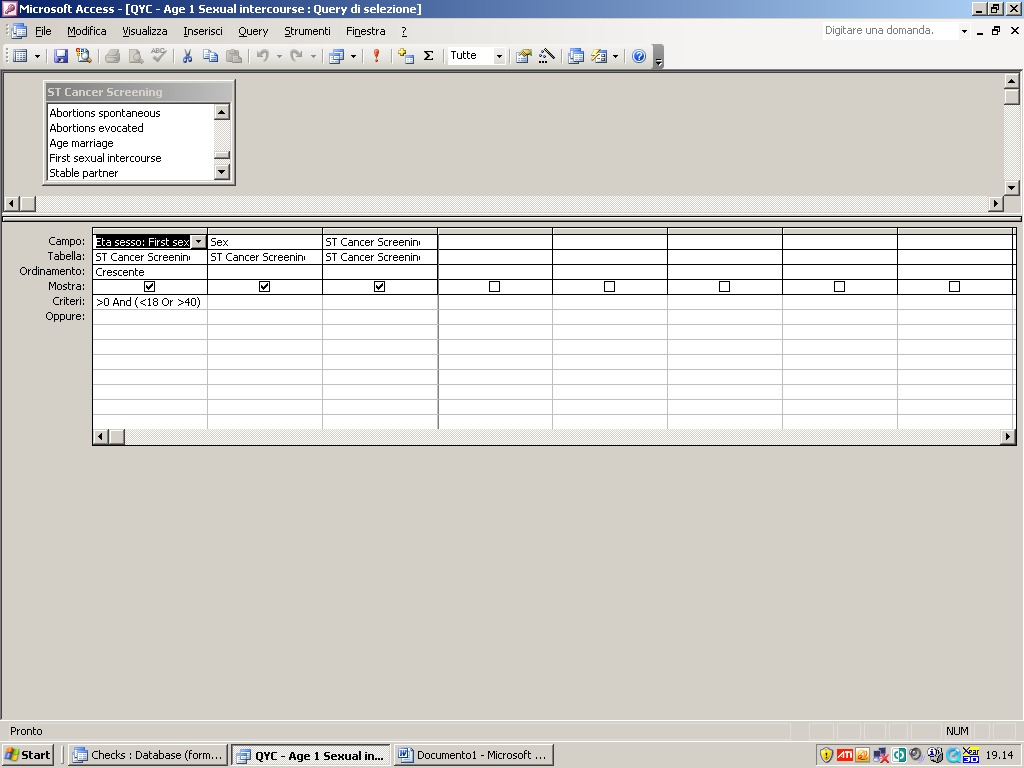


The first sexual intercourse over 40 years and under 18 have can be a data – entry – mistake.

The field “sex” is shown in order to verify if there hasn’t been a mistake in the data entry. (Male is infact a not suitable value because this section of the questionary is limited to female)

### Body Mass Index

QUERY NAME QYC – BMI

FROM TABEL ST Cancer screening

SELECTED FIELD BMI

FILTER: BMI < 17 OR > 41

OTHER SWHOWN FIELDS - - -


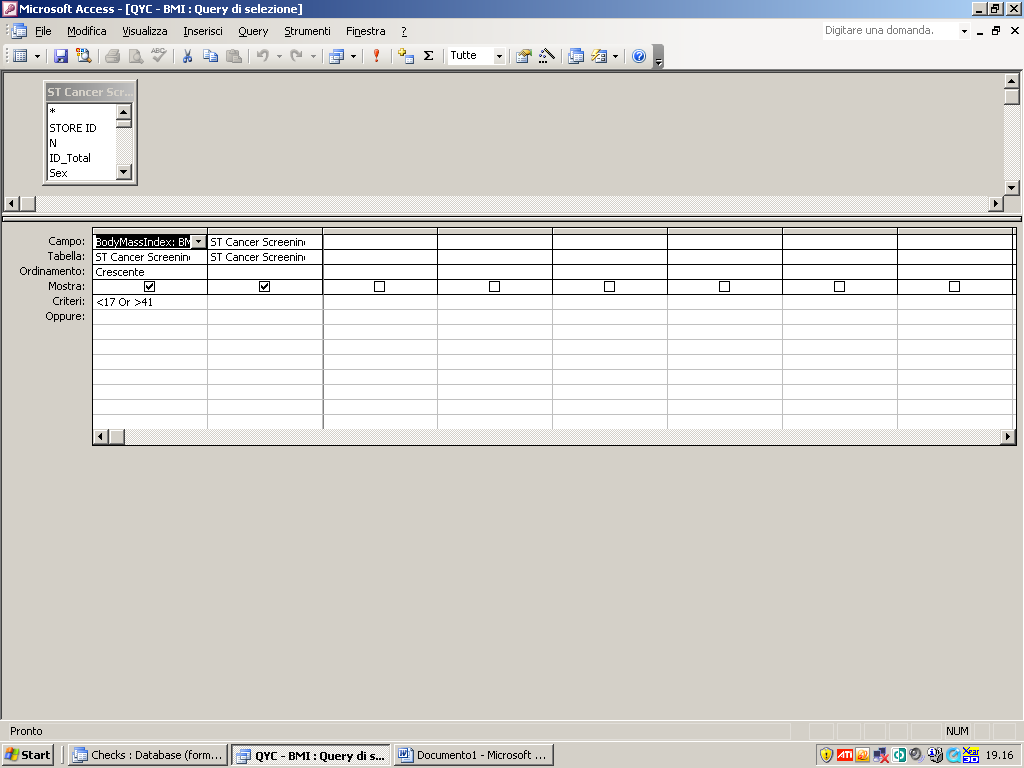


The BMI’s value shows possible mistakes in height and weight

### Height

QUERY NAME QYC – Height

FROM TABEL ST Cancer screening

SELECTED FIELD height

FILTER: height < 140 OR > 195

OTHER SWHOWN FIELDS - - -


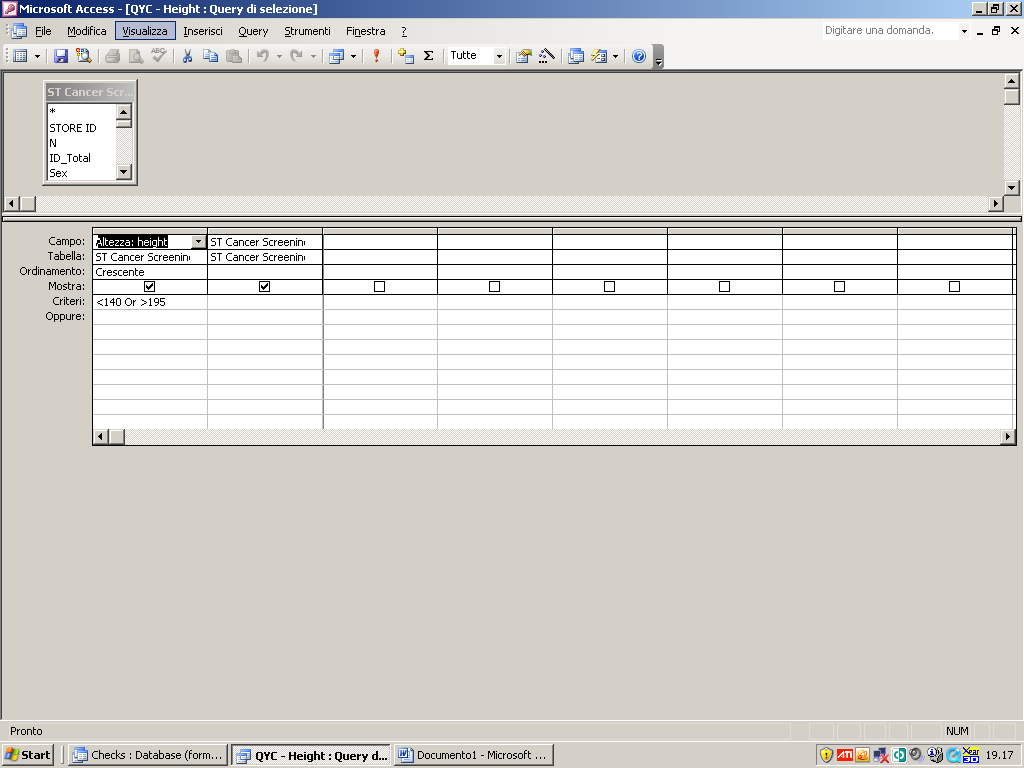


### Number of parturitions

QUERY NAME QYC – N parturition

FROM TABEL ST Cancer screening

SELECTED FIELD N parturitions

FILTER: N parturitions > 6

OTHER SWHOWN FIELDS Sex


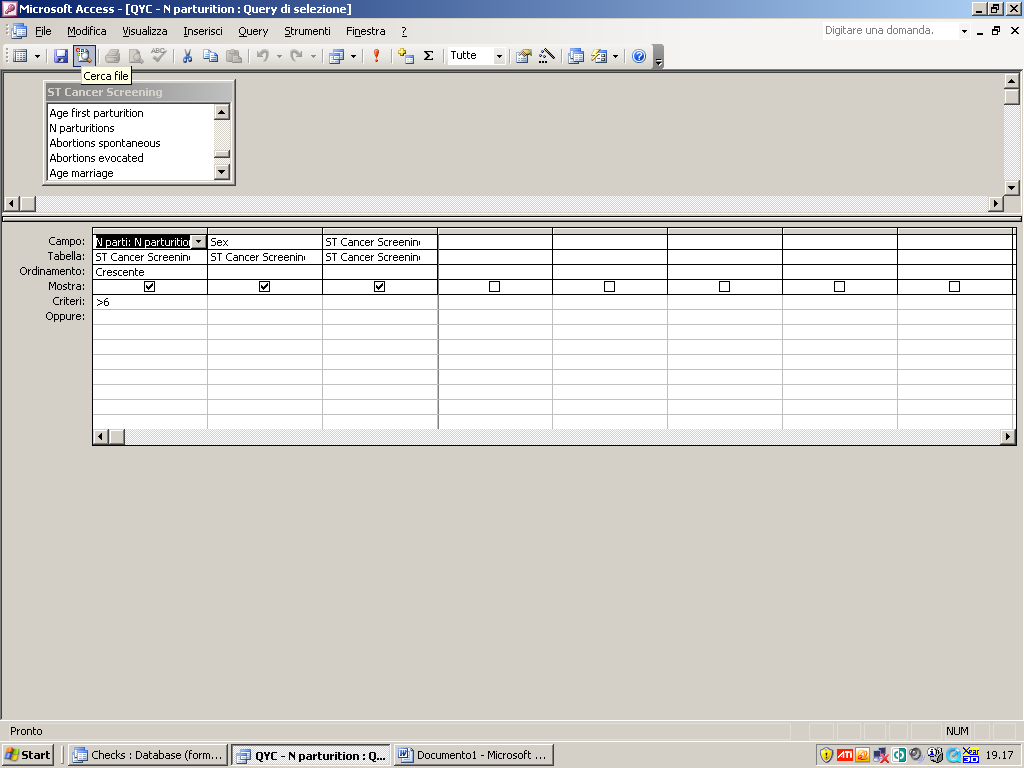


The field “sex” is shown in order to verify if there hasn’t been a mistake in the data entry. (Male is infact an impossible value).

### Number of sons

QUERY NAME QYC – Sons

FROM TABEL ST Cancer screening

SELECTED FIELD Sons

FILTER: sons > 7

OTHER SWHOWN FIELDS - - -


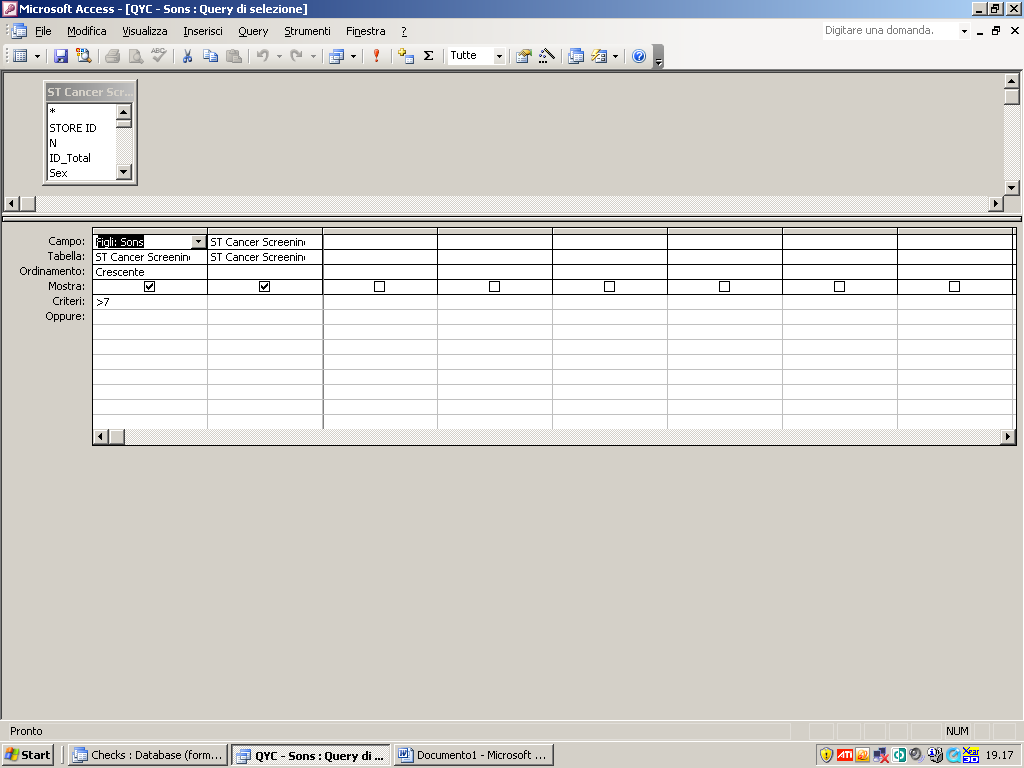


### Weight

QUERY NAME QYC – Weight

FROM TABEL ST Cancer screening

SELECTED FIELD weight

FILTER: weight < 40 OR > 120

OTHER SWHOWN FIELDS - - -


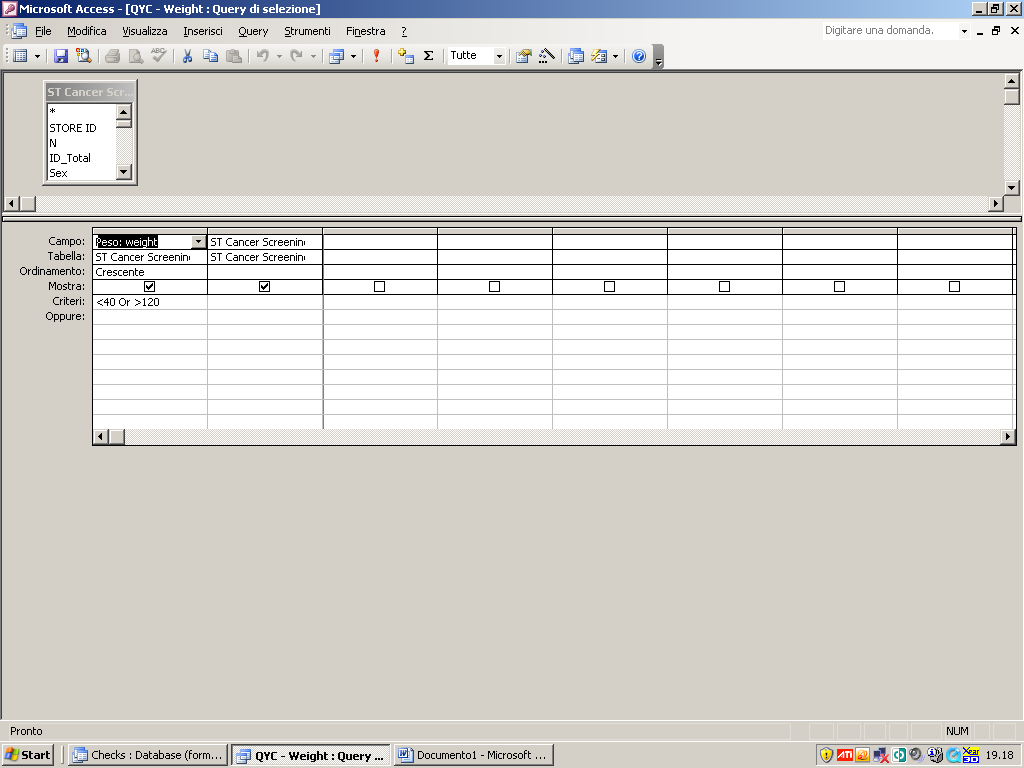

Supplement: Additional file 1 — electronic controls. The data provided represent the methodology used and the filters employed in electronic controls. [file 1471-2288-8-66-S1.doc]
